# “Ready for the future?” – Status of national and cross-country horizon scanning systems for medicines in European countries

**DOI:** 10.3205/000307

**Published:** 2022-03-31

**Authors:** Sabine Vogler

**Affiliations:** 1 WHO Collaborating Centre for Pharmaceutical Pricing and Reimbursement Policies, Pharmacoeconomics Department, Gesundheit Österreich (GÖG/Austrian National Public Health Institute), Vienna, Austria

**Keywords:** pharmaceutical preparation, health technology, horizon scanning, forecast, preparedness, affordability, access to medicines, Health Technology Assessment (HTA)

## Abstract

**Background:** Horizon scanning aims to systematically identify upcoming health technologies and thus allows policy-makers to be better prepared for the entry of new medicines with possibly high price tags into the national health system. The aim of this study is to survey the existence of national and cross-national horizon scanning systems for medicines in European countries.

**Methods:** Experts working in public authorities (members of the Pharmaceutical Pricing and Reimbursement Information/PPRI network) in the WHO European region participated in surveys in 2014 and 2019 and informed about the status of horizon scanning in their country (response rate: 14 and 44 countries, respectively). Identified advanced horizon scanning systems as of 2019 were further investigated based on a literature review.

**Results:** In 2019, six countries (Iceland, Italy, the Netherlands, Norway, Sweden, United Kingdom) reported systematic use of horizon scanning for some new medicines, and four countries (Austria, Denmark, France, Ireland) had some horizon scanning activities ongoing. No systematic use of horizon scanning was reported from the remaining 34 countries. The findings of the survey undertaken five years earlier were similar, with even fewer systems in place. A recent development is the establishment of cross-country initiatives of governments that aim, among others, to jointly perform horizon scanning; the International Horizon Scanning Initiative (IHSI) initiated by the Beneluxa collaboration is the most advanced undertaking in this respect. Countries with systematic use tend to have horizon scanning fully integrated in a system for the management of new medicines, and they use horizon scanning outcomes to inform decisions as to whether or not a Health Technology Assessment will be conducted and price negotiations be started. Differences between existing horizon scanning systems mainly concern the timings of scanning and reporting, the sources for the inputs and the accessibility of the findings.

**Conclusion:** There appears to be a discrepancy between the perceived importance of horizon scanning based on some eye-opening examples in the past and its actual implementation in European health systems. The latter is likely attributable to horizon scanning being resource-intensive. The establishment of new national and international horizon scanning systems offers the opportunity to investigate their impact on sustainable access to affordable medicines from the start.

## Zusammenfassung

**Hintergrund:** Dank Horizon Scanning, der systematischen Recherche über neu auf den Markt kommende Gesundheitstechnologien, sind Entscheidungsträger/innen besser auf den Eintritt neuer, möglicherweise hochpreisiger Medikamente in ihr Gesundheitssystem vorbereitet. Ziel dieser Studie ist es, bestehende nationale und länderübergreifende Horizon-Scanning-Systeme für Medikamente in europäischen Ländern zu untersuchen.

**Methodik:** Expertinnen und Experten von Behörden (Vertreter/innen des „Pharmaceutical Pricing and Reimbursement Information“/PPRI-Netzwerks) in der WHO-Region Europa nahmen an Erhebungen in den Jahren 2014 und 2019 teil und gaben dabei Auskunft über den Umsetzungsstand von Horizon Scanning in ihrem Land (Antworten aus 14 bzw. 44 Ländern). Informationen zu gut ausgebauten Horizon-Scanning-Systemen (Stand: 2019) wurden mittels Literaturrecherche ergänzt.

**Ergebnisse:** 2019 nutzten laut eigener Angabe sechs Länder (Island, Italien, Niederlande, Norwegen, Schweden und das Vereinigte Köngreich) Horizon Scanning systematisch, und in vier Ländern (Dänemark, Frankreich, Irland und Österreich) kam Horizon Scanning punktuell zum Einsatz. In den übrigen 34 Ländern fand Horizon Scanning keine Anwendung. Die Ergebnisse der Erhebung des Jahres 2014 waren ähnlich; allerdings waren damals Horizon-Scanning-Systeme noch seltener verbreitet. Neu ist die länderübergreifende Kooperation von Staaten, bei denen auch gemeinsam zu Horizon Scanning gearbeitet wird; als führendes Beispiel gilt hier die International Horizon Scanning Initiative (IHSI) der Beneluxa-Zusammenarbeit. Länder mit Horizon-Scanning-Systemen zeichnen sich im Allgemeinen dadurch aus, dass diese einen fixen Bestandteil des Arzneimittelsystems, konkret für den Umgang mit neuen Medikamenten, darstellen und eine wichtige Entscheidungsgrundlage dafür bieten, ob in der Folge eine umfangreiche Bewertung mittels eines Health Technology Assessments durchgeführt wird und/oder Preisverhandlungen aufgenommen werden. Horizon-Scanning-Systeme unterscheiden sich zwischen den Ländern hinsichtlich des Zeitpunkts des Scans und der Berichte, der Quellen für einfließende Informationen und der Veröffentlichung der Ergebnisse.

**Schlussfolgerung:** Obwohl Horizon Scanning angesichts einiger in der Vergangenheit prägender Erfahrungen als durchaus wichtig erachtet wird, ist die tatsächliche Umsetzung in den europäischen Gesundheitssystemen derzeit sehr spärlich. Dies mag auch daran liegen, dass Horizon Scanning ressourcenintensiv ist. Die Schaffung neuer nationaler und internationaler Horizon-Scanning-Systeme bietet die Chance, diese von Anfang an mit einer Evaluation über ihre Auswirkungen auf nachhaltigen Zugang zu leistbaren Medikamenten zu begleiten.

## Introduction

Equitable patient access to affordable and effective medicines is a challenge in Europe and globally. Millions of people in low- and middle-income countries (LMIC) have ever since been struggling to afford medications for themselves and for people they care for, which resulted in catastrophic household spending [1], [2], [3]. In addition, the last decade has seen the challenge of sustainable and fair access to medicines high on the political agenda, and the debate has been driven by high-income countries with solidarity-based health care systems [4], [5], [6], [7], [8].

This is, to a major extent, attributable to the market entry of new medicines with (very) high price tags, for instance to treat cancers [9], [10], [11]. While some new medicines may offer major therapeutic progresses, others do not live up to the expectations and cannot offer substantial (added) therapeutic value [12], [13], [14].

To ensure the financial sustainability of publicly funded health care systems, governments have been applying pricing and reimbursement policies. They are supplemented by Health Technology Assessments (HTA) to help select and prioritise cost-effective treatments [15], [16], [17], [18]. Furthermore, demand-side measures which target health professionals and patients such as generic substitution have been implemented with the aim to foster the uptake of lower-priced off-patent medicines [19], [20]. From what is known so far, overall these policies have proven to be successful with regard to defined policy aims such as cost-containment, inclusion of medicines in a benefit’s package scheme and promotion of competitiveness in the off-patent markets compared to non-interventions [21], [22]. However, since they have not yet been sufficiently effective in ensuring sustainable and equitable access to affordable medicines, new policy options are being explored.

In this respect, a kind of a turning point was the hepatitis C medication sofosbuvir, which had come rather unexpectedly in 2014, with a very high price tag. Policy-makers openly admitted that they had not been prepared for this new treatment option [23]. In response, several governments called for mechanisms to inform authorities and public payers long in advance of what is in the pipeline, to allow for strategic preparation [4], [24]. This widespread interest in early alert systems is like a second birth for horizon scanning, which is not at all new.

Horizon scanning is defined as “systematic identification of health technologies that are new, emerging or becoming obsolete and that have the potential to effect health, health services and/or society” [25]. It is based on a standardised methodology that usually consists of five sequential components (identification, prioritisation, early assessment, dissemination, monitoring) [26]. An emerging health technology in this context is a health technology that has not yet been adopted within the healthcare system (e.g. a medicine in the phase II or III clinical trial, or pre-launch stage, a medical device in the pre-marketing stage), and a new health technology is a health technology that is in the launch, early post-marketing, or early diffusion stages [25]. Researchers and public agencies have been working for decades on identifying and assessing new health technologies, including medicines, and since 1997, representatives from Denmark, the Netherlands, Spain, Sweden and the United Kingdom (UK), with associated representatives from Canada and Switzerland have been collaborating on the exchange of information on the safety and efficacy of the new technologies. Started as a working group, it became the EuroScan International Network hosted by the University of Birmingham in 1999, and in 2017 the legal scientific association of EuroScan International Network registered in Germany was founded [27].

Policy-makers aim to be informed early enough before new medicines enter the market so that they have sufficient time to take evidence-based strategic decisions and prioritisation, and call for horizon scanning systems.

While there is increasing debate on the need for horizon scanning, there is limited up-to-date knowledge on the current status of the establishment of horizon scanning systems for medicines in the pharmaceutical policy frameworks in European countries.

Against this backdrop, this article surveys governments’ initiatives of national and cross-national horizon scanning and early alert systems for medicines with a view to exploring developments over years as well as similarities and differences between existing systems.

## Methods

The scope of the study is a system that allows policy-makers to be informed on upcoming medicines (i.e., a horizon scanning or early alert system). Horizon scanning systems of interest could be national ones or cross-country initiatives. Horizon scanning for other health technologies (e.g. medical devices) was not the focus of this research. The study was targeted at the World Health Organization (WHO) European region which comprises 53 countries (including also Central Asian countries).

**Figure 1:**
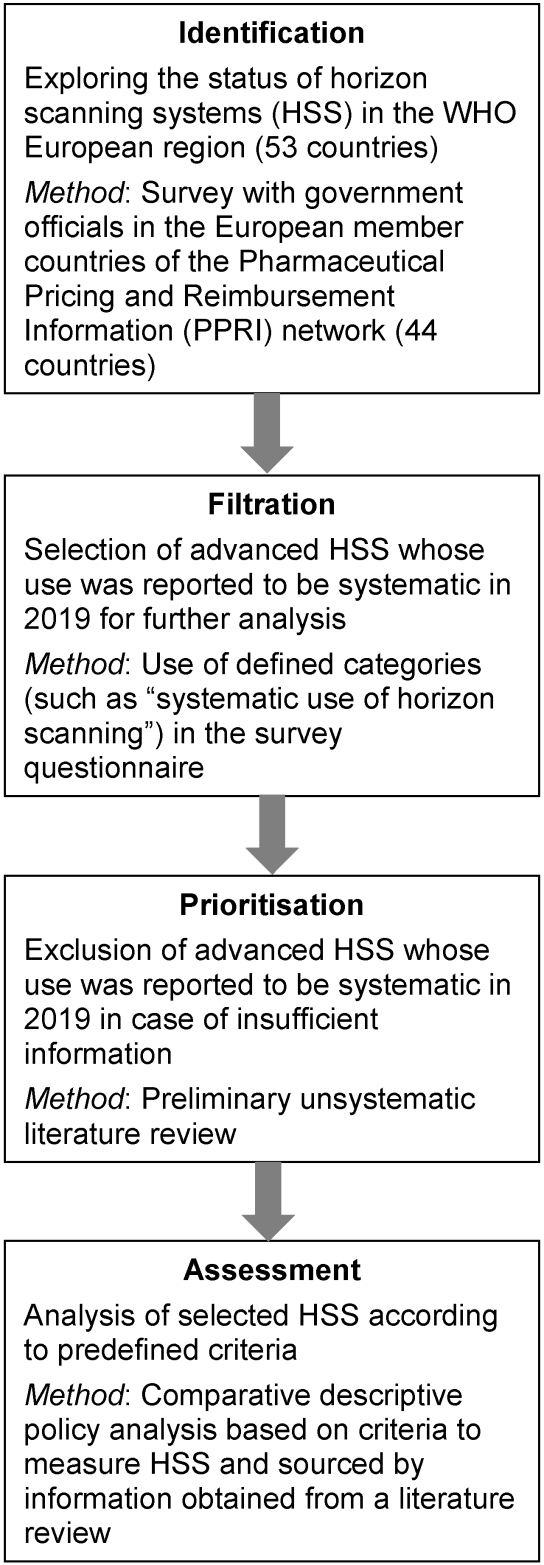
Methods to identify, select and analyse national horizon scanning systems for the scope of this study

As in a horizon scanning process, the aim was to first scan a large number of countries and to identify horizon scanning systems, prioritise and select a few, based on defined criteria, and comparatively analyse them. To do so, a mixed methods approach was used (Figure 1 (Fig. 1)).

Surveys addressing officials in authorities responsible for pharmaceutical pricing and reimbursement in European countries were conducted at two points in time to learn whether or not (advanced) horizon scanning systems for medicines were in place. In 2014 and in 2019, members of the Pharmaceutical Pricing and Reimbursement Information (PPRI) network were addressed to participate in a survey. Both surveys were part of a larger questionnaire that studied further pricing and reimbursement policies for medicines. PPRI is a network of representatives of competent authorities for pharmaceutical pricing and reimbursement of medicines in mainly European countries [28]. At the time of the 2019 survey, the PPRI network comprised 47 countries, thereof the then 28 Member States of the European Union (EU), 16 further countries in the WHO European region (Albania, Armenia, Belarus, Iceland, Israel, Kazakhstan, Kosovo, Kyrgyzstan, Moldova, North Macedonia, Norway, Serbia, Russian Federation, Switzerland, Turkey and Ukraine) and three non-European countries (Canada, South Africa and South Korea). In 2014, the PPRI network comprised 42 countries, thereof 39 countries in the European region. As the study was targeted at European countries, the three countries which are not situated in the European region were excluded from this study. 26 of the then 38 PPRI countries in the European region responded to a questionnaire on policies in 2014, and 14 of them answered the question on horizon scanning. For the second survey of December 2019 with the then 44 PPRI countries in the European region, a slightly different methodology was applied. We prefilled a questionnaire with information that PPRI network members had provided and validated in a 2018 survey, and we asked for validation. 26 countries validated the 2019 data, while for the remaining 18 countries, validated information relating to the year 2018 is available.

Systematically used national horizon scanning systems as of 2019 were selected as case studies if a sufficient level of information was available to allow for meaningful description.

International horizon scanning systems in Europe were identified through a review of cross-country collaborations of European governments in pharmaceutical policy and subsequent contacts to the representatives of collaborations with horizon scanning activities (or plans) in summer 2018 and – to obtain updated information – again in autumn 2019.

The description of national and cross-national horizon scanning systems is based on a review of published literature, including grey literature such as website information, presentations and technical reports.

## Results

### Overview of horizon scanning systems 

In 2019, six countries of the WHO European region (Iceland, Italy, the Netherlands, Norway, Sweden – only inpatient sector, UK) reported systematic use of horizon scanning for some new medicines. Four further countries (Austria, Denmark, France, Ireland) had ongoing horizon scanning activities, however not in a comprehensive, integrated manner in the health system, and this was also the case for the outpatient sector in Sweden. In the remaining 34 countries, no use of horizon scanning as part of the pharmaceutical policy framework was reported; however, Belgium informed on plans to introduce a national horizon scanning system.

Five years earlier, two countries (Iceland and Norway) informed about an ongoing implementation of a national horizon scanning system, while Italy and UK already reported their up and running projects in 2014 (partially missing information for some countries).

At cross-country level, some collaborations (e.g. Nordic Pharmaceutical Forum, Valletta Declaration) plan to work jointly on horizon scanning. At the time of writing (Q2/2020), the sole cross-country system established is the International Horizon Scanning Initiative of the Beneluxa Initiative. Its members are, to some extent, countries (e.g. the Netherlands, Norway) that already have a national horizon scanning system (Table 1 (Tab. 1)).

### Details of national horizon scanning systems with systematic use

National horizon scanning systems of the countries that reported systematic use (except Iceland) are described in further detail in the following (comparative overview in Table 2 (Tab. 2)).

One of the oldest horizon scanning systems is the Italian Horizon Scanning Project [29], [30], [31], [32]. Established in the Veneto Regional Health Unit in 2006, it aims to inform payers (the Veneto region and other regions) and the national Medicines Agency AIFA (Agenzia Italiana del Farmaco) on upcoming medicines as well as medical devices with medicated coating. It offers three different types of reports at different timings: 36 months before the potential market authorisation granted by the European Medicines Agency (EMA), a report is submitted to AIFA that mainly contains information on upcoming medicines, phase II trial data and the indications of ongoing phase III trials. 18 months before possible EMA authorisation, a report mainly for internal purposes assesses available results of the first phase III completed trials. A New Product Information report is delivered twelve months before possible EMA authorisation, and it critically assesses available data on efficacy and safety of the new medicines versus current standards [29], [33]. Data are made available in the CINECA platform which is a closed platform of Italian regions [34].

Since 2012, the Netherlands have made use of horizon scanning. In the first years, it used to be a rather “light horizon scanning system” [34] and mainly relied on the results of other horizon scanning institutions such as the US Agency for Healthcare Research and Quality (AHRQ). In 2017, Horizonscan+ was launched, and the Ministry of Health tasked the umbrella organisation for the health insurers, the National Healthcare Institute (ZINL), to coordinate this expanded system. Horizonscan+ aims to identify new potentially innovative medicines for outpatient and inpatient use two years ahead of marketing authorisation. It also aims to learn about expected list prices and volumes of these new medicines as well as indication extensions of existing innovative medicines [35]. Information is publicly accessible through a dedicated ZINL website in Dutch [36].

Norway established the “National System for Managed Introduction of New Health Technologies within the Specialist Health Service” (“Nye Metoder”) in 2013 [37]. It aims to ensure a consistent process for all new health technologies, including medicines and medical devices, in hospital care. A key component is the systematic use of health technology assessments for all new health technologies in different formats (either mini-HTA done by hospitals, single technology assessment against a comparator done by the Norwegian Medicines Agency, or full HTA to compare various methods performed by the Norwegian Institute of Public Health). A horizon scanning notification (or a proposal of a stakeholder) guides a committee of representatives from the federal state and regions (payers) on their decision about the type of HTA to be performed. The Norwegian Medicines Agency prepares the horizon scanning notifications for medicines, assisted by literature reviews provided by the Norwegian Institute of Public Health [37], [38], [39], [40]. Medicines and other “methods” identified in the horizon scanning exercise are published in an open access database (MedNytt) of the Norwegian Institute of Public Health, which is updated ten times a year [40], [41]. Since January 2018, findings of horizon scanning relating to medicines for outpatient use can also result in HTA [38].

Similar to the processes in the Netherlands and Norway, horizon scanning in Sweden is integrated in the process of managed introduction of new medicines in hospitals. It aims to inform the regions who are the payers, by providing a basis for their decision on whether or not a “managed introduction” based on a full HTA or company collaboration is needed for a specific medicine. Horizon scanning is done by the four largest regions (Västra Götaland, Östergötland, Stockholm and Skåne), through the Horizon Scanning working group (one representative of each of the four regions and a coordinator), as part of the “Collaboration Model” framework coordinated by the overarching Swedish Association of Local Authorities and Regions [34], [42]. The working group members regularly monitor sources, such as international and national agencies. Industry input is gained through pipeline hearings and scanning of websites [43]. The Horizon Scanning working group prepares the first step of the filtration process of the aggregated data at least one to two years before marketing authorisation. Filtration is finalised by the decision on whether or not an early assessment report (“tidiga bedömningsrapporter”), which describes the current state of knowledge, will be produced (available around six months before marketing authorisation) [43], [44]. Filtration is informed by inputs of clinical experts of the counties and considers several criteria [42], [45]. Outcomes of horizon scanning such as early assessment reports and medicines identified for early assessment reports are published on the website of the Health and Medical Administration of the Stockholm region [46].

The UK has a long history of horizon scanning, and UK’s countries have their own systems in place. In England, horizon scanning is the responsibility of the Horizon Scanning Research and Intelligence Centre of the National Institute for Health Research (NIHR HSRIC), which was called National Horizon Scanning Centre (NHSC) until 2012. Established as an independent research team at the University of Birmingham in 1998, it was incorporated as a research programme within the NIHR in 2006 [47]. It provides an advance notice of new and emerging health technologies and interventions that are likely to have a significant impact on the National Health Service (NHS) and/or on patients within the next two to three years. The key customer is National Institute for Health and Care Excellence (NICE), which expects to receive a brief report on new products at least 20 months before launch. Two major approaches are applied in the identification process: a general routine identification through a “horizontal scan” and an in-depth scanning and review in defined areas with known multiple or complex developments and for patient groups with significant or unmet needs [34]. In addition to publications (e.g. scientific journals, other horizon scanning organisations), the key information sources are “pipeline meetings” held with industry and the UK PharmaScan database. This database was established by the Department of Health and the Association of the British Pharmaceutical Industry, as a commitment in the 2009 Pharmaceutical Price Regulation Scheme to establish a single, unified horizon scanning process to identify new technologies in development by the industry [48]. Operational since 2010, it was funded by the Department of Health and horizon scanning bodies and is hosted by NICE. Access to UK PharmaScan, which is expected to be fed by pharmaceutical companies, is highly restricted to a few institutions such as NICE, NIHR HSRIC, further horizon scanning institutions in Wales, Scotland and Northern Ireland and UK Medicines Information [49], [50], [51]. The latter provides a horizon scanning service by offering reports such as “Prescribing Outlook” to health care professionals and NHS budget holders (limited access for those working in the NHS) [34]. Scotland and Wales have separate, partially complementary horizon scanning systems performed by the HTA institutions Scottish Medicines Consortium (SMC) [52] and All Wales Medicines Strategy Group (AWMSG) [53], respectively. 

### International Horizon Scanning Initiative (IHSI)

The International Horizon Scanning Initiative (IHSI) was established in October 2019 as an independent legal entity that will provide outcomes to its member countries. It was initiated by the Beneluxa Initiative, a cross-national collaboration of five European countries (Belgium, the Netherlands, Luxembourg, Austria, Ireland), but as a separate area for collaboration independent from the status of being a Beneluxa member. The Beneluxa Initiative invited further countries to participate in collaborating in horizon scanning, without having to join the Beneluxa Initiative [54]. IHSI started with eight countries: Belgium, the Netherlands, Denmark, Ireland, Norway, Portugal, Sweden, Switzerland, and further countries expressed interest [55].

In preparation for IHSI, the Belgian Health Care Knowledge Centre (KCE) was commissioned to develop a model for a joint horizon scanning system and to assess the feasibility of the proposed methodology for Belgium. KCE recommended to set up a central horizon scanning unit (either to be newly established or as an expansion of the horizon scanning activities of an existing unit), to provide sufficient resources, to pilot the proposed methodology and to perform an evaluation with subsequent adjustments [34].

At the end of 2018, an open market consultation was conducted to inform market operators about an upcoming public procurement procedure for setting up an international horizon scanning system and to obtain market operators’ input on the viability of the procurement design and conditions [56].

In Q1/2020, a tender was launched with the aim to select a third party provider tasked with building and implementing the IHSI Joint Horizon Scanning Database, which will consist of two key elements: the technical infrastructure to host the database, and a scientific component relating to the data collection exercise which is intended to continuously populate the database. At the time of writing (Q2/2020), the database was expected to be up and running by end of 2020, and first datasets were expected at the beginning of 2021 [57].

## Discussion

To the author’s knowledge, this is the first survey on the existence of national and cross-national horizon scanning systems throughout the WHO European region. Previous studies – some of which were performed more than 15 years ago – only considered existing horizon scanning systems, usually with a focus on specific aspects, such as selection or search methodologies [32], [58], [59]. This study, which applied a wide geographic scope, showed that national systems with systematic use of horizon scanning for emerging health technologies are in place in a rather limited number of countries. As a trend, these are high-income countries that have also been exploring a range of other policy options along the value chain [28]. As horizon scanning systems are resource-intensive and their establishment and maintenance require capacity of experts and sufficient funding, it is not surprising that higher-income countries were among the first ones to establish these systems. They could also build on horizon scanning activities performed earlier by research and HTA institutions such as in the frameworks of the European Network for Health Technology Assessment (EUnetHTA) project or the EuroScan collaboration.

At the same time, there appears to be high interest of numerous countries in an early alert of what is in the pipeline. One option to gain access to horizon scanning outcomes in the case of limited capacity and resources is through collaborative efforts: Several countries (members of the Beneluxa Initiative and beyond) joined the newly launched International Horizon Scanning Initiative. Further cross-country collaborations, which were established in recent years (e.g. Valletta Declaration, Nordic Pharmaceutical Forum), are committed to work together on horizon scanning but have not yet started implementation; interestingly, some member countries of the mentioned cross-country collaborations also joined IHSI. Collaborative efforts help containing the costs, which may be substantial: in the 2017 feasibility study for a collaborative horizon scanning system of the Beneluxa Initiative, which then resulted in establishing the IHSI, an annual budget amounting to eight to ten full-time equivalents was established [34].

Countries with advanced horizon scanning systems tend to establish them in an integrative manner in the respective pharmaceutical policy framework: As the country examples of this study show, horizon scanning is applied as a first, fundamental step in the system for managing the entry of new, possibly high-priced medicines in the national health service or social insurance system. The findings of systematic application of horizon scanning are used, frequently in a standardised way, to decide on the further path of identified medicines, i.e. whether or not a full HTA will be performed, price negotiations will be launched and/or managed-entry agreements will be concluded. It is obvious that budget impact considerations play a role, and this review confirmed that they are explicitly or implicitly used for the filtration and prioritisation process, in addition to other criteria.

To contain the cost for the establishment and maintenance of horizon scanning systems, countries could limit their scope (e.g. on oncology medicines in Austria [60], [61], [62]), or focus the identification strategies on defined therapeutic areas. An established horizon scanning system which has developed a methodological basis can rather easily extend its scope if there is need and/or resources are provided. For instance, the Austrian HTA institution (named Austrian Institute for Health Technology Assessment since 2020), which has managed the Horizon Scanning System in Oncology since 2007, established a Horizon Scanning System for COVID-19 medicines and vaccines in Q1/2020 and published the first report in April 2020.

The study findings point to the importance of smooth coordination and well-functioning communication between technical experts and policy-makers if horizon scanning shall support the managed entry of new medicines. Some authors see an enhanced role of the HTA community to provide for a better bridging [63]. Furthermore, in order to ensure that information actually reaches the policy-makers, optimised information flows and deliverables are needed. In fact, some of the studied horizon scanning systems have defined deliverables targeted at policy-makers (e.g. brief new product information reports) while some other reports are rather internal (e.g. Italy). In addition, a well-defined path, such as in the Netherlands and Norway, to manage the introduction of new medicines with deadlines for submitting the reports and scheduled meetings, in which a committee considers the horizon scanning results, likely enhances policy relevance. If horizon scanning is done by research groups that are not connected to the “system” (though publicly funded), there may be the risk that the outcomes will not be considered in policy decisions. This would constitute a waste of resources, and ignorance of the work by the policy-makers is frustrating for the experts who did the scanning. In England, an independent academy-based research group on horizon scanning was integrated in an NHS institution in 2006 [47], which may have strengthened its policy relevance.

Besides coordination and cooperation, Norway considers transparency as another success factor of their “New Methods” system [64]. Regarding the publication of outcomes, the studied countries apply different approaches: while Norway, Sweden and the Netherlands present their findings on publicly accessible websites and thus support information exchange among countries, Italy and UK share the findings only with professionals and experts in their NHS system. In contrast, the newly launched International Horizon Scanning Initiative aims to solely use publicly accessible information, and this would allow their founders to publish it without limitation, should they decide to do so [65]. At national levels, with regard to the confidentiality level of the data sources, different approaches were identified between the countries: While some countries considered solely published information, others combined open access information with industry input. The latter was, among others, gained in “pipeline meetings” with pharmaceutical companies that are held in Sweden and the UK. In addition, the UK established the UK PharmaScan database. However, its benefit of providing a combined data source for the different countries of the UK is likely undermined by its dependency on the pharmaceutical companies’ willingness to feed the database. It was reported that frequently, companies need prompting to complete the database and that not all companies are registered users [34]. In particular, it is interesting to note that the Department of Health funds UK PharmaScan, but does not have access to it [51].

Finally, horizon scanning is usually targeted at identifying emerging new, possibly innovative health technologies, including medicines. However, for understanding the market, it is also important to know when (less expensive) alternatives will be launched and can contribute, due to competition, to savings in public budgets. Obtaining information on the date of the patent expiry is another challenge for governments. In this respect, the emergence of therapeutic equivalents could constitute an added value of horizon scanning systems. The English system, for instance, also scans for first biosimilar medicines of the indication [34].

The study has some limitations. The PPRI surveys were focused on identifying the existence of horizon scanning systems, but did not investigate details of existing systems. Thus, further sources had to be consulted.

The response rate to the respective question on the existence of a horizon scanning system was rather low in the 2014 survey, which overall limits the meaningfulness of this survey.

Despite its systematic use, the Icelandic horizon scanning system could not be included in the more detailed comparative description due to lack of data.

The WHO European region consists of 53 countries. Given the difficulties to obtain information from some smaller countries and Central Asian countries (which are also part of the WHO European region), the study was limited to the 44 PPRI countries in the European region (missing countries include mini states such as Andorra, Liechtenstein or Monaco and Central Asian countries such as Azerbaijan or Turkmenistan).

While the concept of a (national) horizon scanning system was defined in the surveys, it was up to the respondents to classify ongoing horizon scanning activities as a horizon scanning system. It is acknowledged that other respondents might have considered horizon scanning activities, such as those undertaken by the HTA institution in Denmark [66], as systematic use of horizon scanning.

## Conclusions

The study showed that, while horizon scanning is overall considered as an important policy tool, in particular with regard to contributing to ensuring patient access to new, possibly innovative medicines, only a few European countries actually have an advanced horizon scanning system in place. Systematic use of horizon scanning is resource-intensive, requires expertise in methods and access to data sources, and this may explain the lag in its implementation. Countries with a systematic use of horizon scanning started establishing their horizon scanning system in the last decade or even earlier, and most of them have integrated it into their national pharmaceutical pricing and reimbursement system, with the aim to use the outcomes strategically to inform the next steps such as assessment as well as pricing and reimbursement decisions.

The analysis highlighted that existing horizon scanning systems differ between European countries. The design of the horizon scanning systems varies, among others, with regard to the methodology applied, confidentiality of the data sources and the dissemination strategy. This will, in return, impact the uptake of the findings by other stakeholders in the country and by other countries.

With the establishment of new national horizon scanning systems and the use of the outcomes from cross-country collaborative systems such as the IHSI, it is advised to monitor their performance as well as to share experiences to allow lessons learning for the future. In particular, it is recommended studying the effects of different designs of horizon scanning systems on improving sustainable and equitable access to affordable medicines.

## Notes

### Acknowledgements

Sincere thanks go to the members of the Pharmaceutical Pricing and Reimbursement Information (PPRI) network: experts working in public authorities in several countries who are not mentioned by name in compliance with the PPRI policy.

Furthermore, I thank my colleague Nina Zimmermann for her involvement in the 2014 and 2019 surveys with the PPRI network and for reviewing an earlier draft of this article.

### Funding

The Austrian Federal Ministry of Social Affairs, Health, Care and Consumer Protection has been funding the work of the PPRI Secretariat to manage and maintain the PPRI network.

### Competing interests

The author declares that she has no competing interests.

## Figures and Tables

**Table 1:**
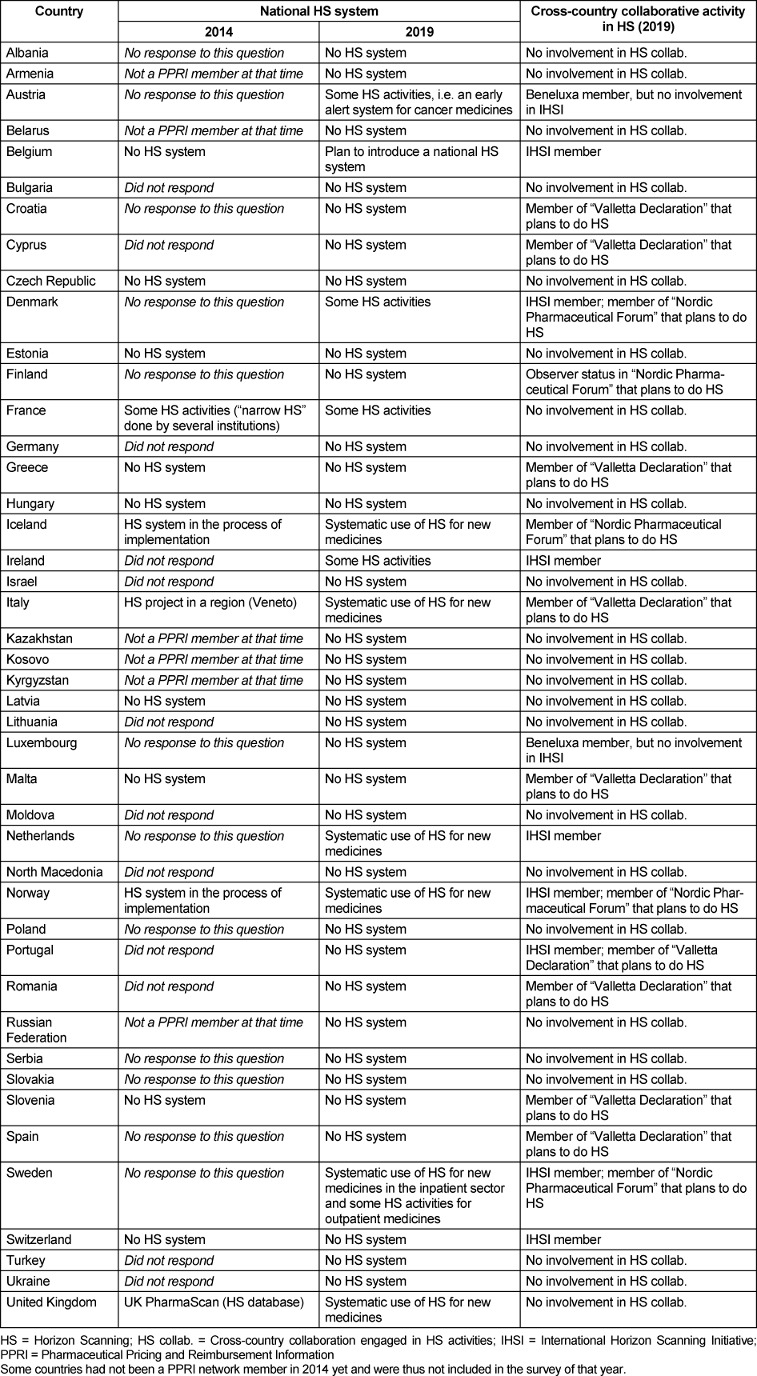
Horizon scanning in the PPRI network member countries in 2014 and 2019

**Table 2:**
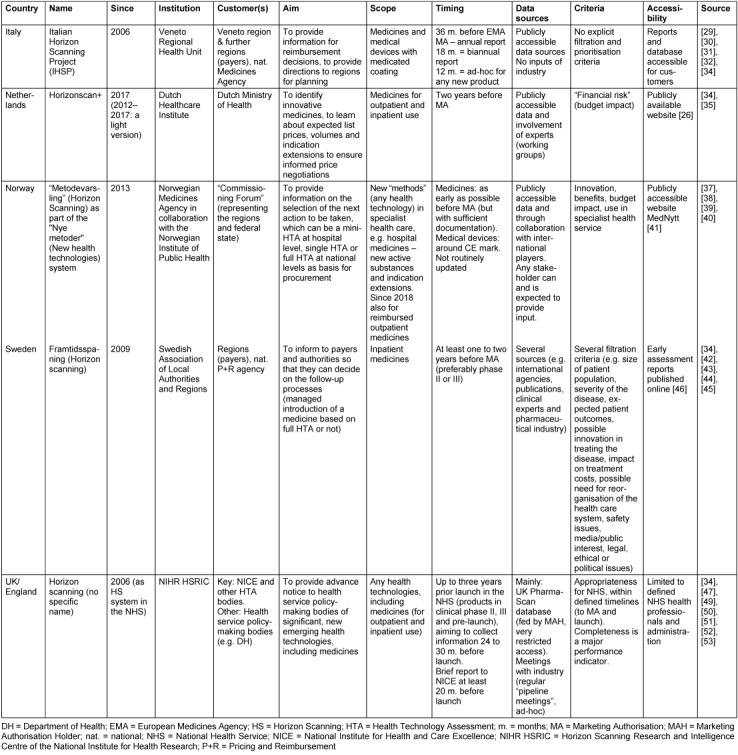
Dimensions of horizon scanning in countries with its systematic use
